# Comparison of the Mid-Term Outcomes of Robotic Magnetic Navigation-Guided Radiofrequency Ablation versus Cryoballoon Ablation for Persistent Atrial Fibrillation

**DOI:** 10.3390/jcdd9030088

**Published:** 2022-03-17

**Authors:** Xiang Li, Yangyang Bao, Kangni Jia, Ning Zhang, Changjian Lin, Yue Wei, Yun Xie, Qingzhi Luo, Tianyou Ling, Kang Chen, Wenqi Pan, Liqun Wu, Qi Jin

**Affiliations:** Department of Cardiovascular Medicine, Ruijin Hospital, Shanghai Jiao Tong University School of Medicine, Shanghai 200001, China; htqqdd@126.com (X.L.); byy12333@rjh.com.cn (Y.B.); jiakangni@126.com (K.J.); dr_ningzhang@163.com (N.Z.); linchangjian222@126.com (C.L.); weiyue0720@gmail.com (Y.W.); xy11992@rjh.com.cn (Y.X.); 18817821284@163.com (Q.L.); lty11498@rjh.com.cn (T.L.); chenkang1978@163.com (K.C.); drpanwenqi@163.com (W.P.)

**Keywords:** atrial fibrillation, catheter ablation, cryoballoon, robotic magnetic navigation

## Abstract

Introduction: Currently, numerous ablation techniques are available for atrial fibrillation (AF), in addition to manual radio frequency ablation. The aim of this prospective, non-randomized concurrent controlled trial was to compare the mid-term efficacy and procedural outcomes of persistent AF (PerAF) using cryoballoon (CB) and robotic magnetic navigation (RMN). Methods: Two hundred PerAF patients were assigned, in a 1:1 ratio, to undergo catheter ablation using RMN (RMN group) or CB (CB group). The primary endpoint was freedom from AF recurrence following a 3-month period after the index ablation. The secondary endpoint was peri-procedural outcomes, including the total procedure time, left atrial procedure time, fluoroscopy time, and fluoroscopy dose. The Two-step cluster analysis was used to determine the efficacy of RMN and CB between the different groups. The Cox proportional hazard model and restricted cubic spline were used to determine predictors for AF recurrence. Results: At the mean follow-up of 28.1 ± 9.7 months, the primary endpoint was achieved in 71 PerAF patients in the RMN group and in 62 PerAF patients in the CB group (71% vs. 62%, *p* = 0.158). Compared with CB, RMN-guided ablation led to a longer procedure time (*p* < 0.001), but with less radiation (*p* < 0.001). Cluster analysis returned two clusters of patients and RMN was favorable for one cluster (*p* = 0.037), in which more patients presented with diabetes mellitus and smaller left atria. Conclusions: For patients with PerAF, CB is generally equivalent to RMN-guided ablation with regard to overall efficacy. RMN-guided ablation could be favorable in specific patient populations presenting with diabetes mellitus and smaller left atria.

## 1. Introduction

Globally, atrial fibrillation (AF) is the most prevalent sustained arrhythmia and represents a major socioeconomic burden. In addition, it is associated with increased morbidity and mortality [1,2]. It is estimated that 15.9 million people will have AF in the United States by 2050 [3] and 17.9 million in Europe by 2060 [4]. Radiofrequency (RF) catheter ablation targeting pulmonary vein isolation (PVI) is a well-established treatment for patients with paroxysmal AF, in view of its superiority to antiarrhythmic drug therapy [5]. However, to date, the traditional manual catheter RF ablation technique still presents challenges. The complicated ablation procedure places a high demand for strategies on physicians. Moreover, the increased procedure duration [6] can lead to compromised safety and efficacy. Fortunately, additional tools and strategies have emerged over the past two decades. Cryoballoon (CB) ablation offers the advantage of delivering the treatment during a single energy application, and thus reduces the requirements for operator skill. Robotic magnetic navigation (RMN)-guided ablation relieves the operator of holding the catheter in place. Therefore, the physician can place greater mental focus on patient monitoring, diagnosis, and treatment, while remaining seated in the control room. In addition, the magnetic catheter is softer, has greater reach, stability [7], and access than a manual catheter. Previous studies have demonstrated the advantages of both of these therapies for certain patients [8,9]. However, few studies have directly compared the two therapies, especially for patients with persistent AF (PerAF). This study was designed to compare the procedural and mid-term outcomes of CB and RMN-guided ablation for patients with PerAF.

## 2. Methods

### 2.1. Patient Population

In this prospective, non-randomized concurrent controlled trial, patients with PerAF were consecutively recruited from Ruijin Hospital, Shanghai Jiao Tong University School of Medicine between June 2016 and October 2019. Patients were considered eligible if they were: (1) 18–75 years old; (2) with PerAF (lasting more than 7 days, but within 5 years) refractory to at least one antiarrhythmic drug (Class I/III); (3) undergoing ablation for the first time. Exclusion criteria were as follows: (1) with history of left atrial surgery; (2) left ventricular ejection fractions (LVEF) ≤ 35%; (3) left atrial diameter (LAD) ≥ 55 mm; (4) with history of myocardial infarction, percutaneous coronary intervention, heart surgery, transient ischemic attack or stroke within 3 months prior to the procedure; (5) with transesophageal echocardiography witnessed atrial thrombus; (6) with uncontrolled hyperthyroidism; (7) expectant mothers; (8) unable to complete a full follow-up visit. Eligible patients were assigned, in a 1:1 ratio, to undergo ablation guided with RMN (RMN group) or by means of CB (CB group). All of the participating patients provided written informed consent. This study was approved by the ethics committee of our institution, in accordance with the principles of the Declaration of Helsinki.

### 2.2. Preparation for Procedure

Antiarrhythmic medications were discontinued prior to the ablation procedure for at least five half-lives. All of the patients received standard anticoagulation therapy for at least 1 month. The international normalized ratio was maintained at 2 to 3 prior to the procedure for the patients receiving warfarin. After transesophageal echocardiography confirmed the absence of atrial thrombus, each patient received an electrophysiological study in conscious state.

### 2.3. CB Ablation

The pulmonary vein inner diameter was measured by computed tomography angiography to determine the size of selected CB. Following transseptal puncture, a CB and Achieve mapping electrode were placed. A hexapolar circular catheter was used for mapping and recording before and after electrical pulmonary vein isolation. A second-generation CB (Arctic Front Advance Cardiac Cryoablation Catheter, Medtronic) was inserted with the use of transseptal puncture and over-the-wire delivery technique. Two CB applications, each 3 min in duration, were applied for each pulmonary vein. Pulmonary vein isolation was confirmed by an entrance block. Adjustment of the application time and necessity of additional freeze were left to the operator’s discretion. During ablation of the right superior and inferior pulmonary veins, the phrenic nerve was continuously monitored by fluoroscopy or pacing, in order to reduce the risk of phrenic nerve paralysis.

### 2.4. RMN-Guided Ablation

As described previously [10], the open-irrigated ablation catheter (NaviStar™ RMT ThermoCool™; Biosense Webster, CA, USA) was connected to a 3D mapping system (CARTO™, Biosense Webster, CA, USA or EnSite™, St. Jude Medical, MN, USA) and the RMN Niobe™ ES system (Stereotaxis Inc., St. Louis, MO, USA) to perform 3D LA electroanatomic mapping and ablation. Additional fracture potential ablation or linear ablation might be performed when necessary. The RF current was delivered for 30−40 s per lesion, applying 30–40 W (irrigation flow rate 17 mL/min) with the generator (Stockert, Biosense Webster, CA, USA) in a power-controlled mode. Power was selected based on the location of catheter tip in the LA. Once PV isolation was achieved, electrical cardioversion was attempted. For patients whose rhythm could not be converted to the sinus rhythm, the LA roof line lesion was created by the RMN catheter or cryoballoon, respectively in either group to ease subsequent electrical cardioversion. Substrate modification, such as ablation of complex fractionated atrial electrogram, was not allowed in this study.

### 2.5. Follow-Up

All of the participant patients remained in the hospital under observation for at least one night. The first outpatient clinic visit was scheduled at 1 and 3 months, and for every 6 months thereafter. Scheduled Holter was the main method for recurrence assessment during follow-up. In addition, patients that have developed symptoms of suspected AF recurrence were advised to undergo an electrocardiogram at the nearest hospital.

### 2.6. Endpoints

The primary endpoint for this study was freedom from AF recurrence during mid-term follow-up. AF recurrence was defined as any of the following events: (1) sustained AF (lasting > 30 s); (2) atrial flutter or atrial tachycardia; (3) prescription of antiarrhythmic drugs (Class I/III); (4) repeat ablation. Any arrhythmia that occurred during a standard 3-month blanking period was not counted as AF recurrence. The secondary endpoint was peri-procedural outcomes, including: (1) procedure duration, defined as the total time from the first venous puncture to sheath withdrawal from the groin, in minutes; (2) left atrial procedure duration, the time from transseptal puncture to sheath withdrawal from the left atria; (3) fluoroscopy time, the total number of minutes the fluoroscopy beam was activated; (4) radiation dose and dose area product.

### 2.7. Statistical Analysis

Statistical analysis was performed with R (R version 4.0.2, R Core Team) and SPSS (IBM SPSS 25.0, SPSS Inc. NT, Chicago, IL, USA). Continuous variables were presented as mean ± SD and categorical variables as numbers and percentages. For intergroup comparison, the student’s *t*-test or Mann−Whitney test was used. Binary variables were assessed by Pearson’s χ^2^ test or Fisher’s exact test where appropriate. Interval-censored Cox regression was performed by Stata (Stata 17.0, Stata Corp, College Station, TX, USA), proportional hazards assumption was accessed prior to the survival analyses, and the results were listed in Supplementary Files. The AF recurrence-free survival rate in RMN and CB groups was assessed by Kaplan−Meier curves and the log-rank test. The Cox proportional hazard model was applied to test the consistency of the group effect, while accounting for patient age, gender, body mass index (BMI), LAD, left atrial volume (LAV), and CHA_2_DS_2_-VASc score. LAV was measured by the contrast computed tomography scan of left atrium. The principal component analysis was intended to obtain components that represent different clinical factors. The scree plot helped in determining the optimal number of components for further Two-step cluster analysis. To determine the risk factors related to AF recurrence, the Cox regression analysis was carried out again. The Cox regression model of restricted cubic spline was used to visualize the association between AF recurrence and patients’ LAV on a continuous scale. The spline was adjusted for patient age, gender, BMI, LVEF, history of diabetes mellitus, hypertension, and ablation technique. A preliminary investigation suggested that three knots were appropriate for spline modeling and the median of the LAV was set as the reference value of the hazard ratio. A *p* < 0.05 was regarded as significant.

## 3. Results

### 3.1. Patient Characteristics

A total of 200 patients who completed their follow-up were enrolled in this study, with 100 assigned in the RMN group and 100 in the CB group. The average and maximum follow-up times were 28.1 ± 9.7 and 36 months, respectively. The participants’ average age was 59.1 ± 9.8 years old and 75% were male. There were no significant differences in baseline characteristics between the two groups (Table 1).

### 3.2. Primary Endpoint

**Figure 1:**
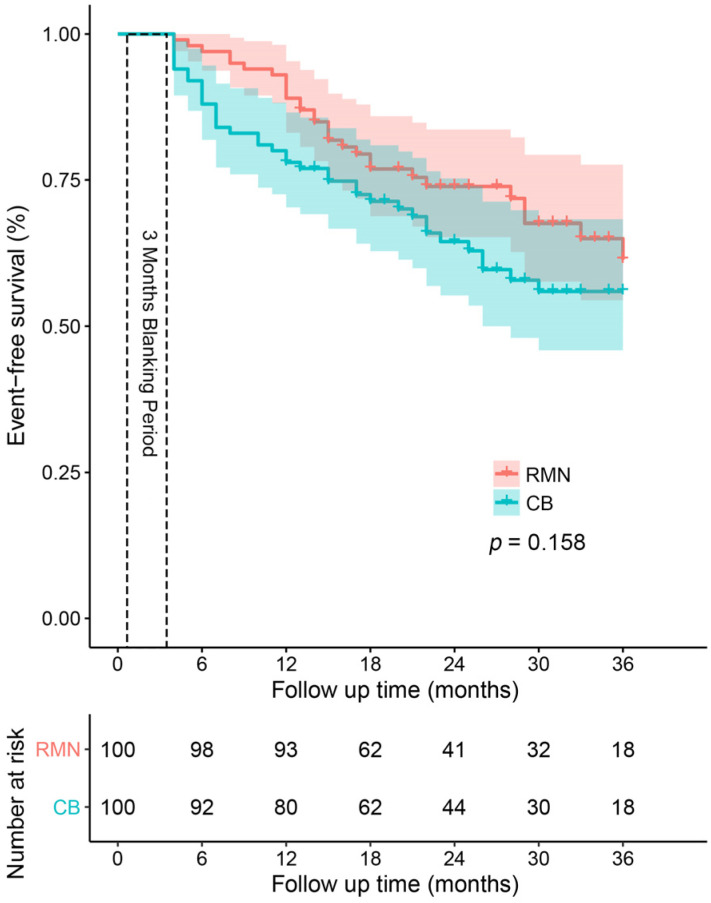
Kaplan−Meier curves showing the cumulative freedom from recurrence of atrial fibrillation after CB and RMN-guided ablation. The freedom from recurrence of AF did not differ between the two groups when compared by the log-rank test (*p* = 0.158). RMN: Robotic magnetic navigation; CB: Cryoballoon.

**Figure 2:**
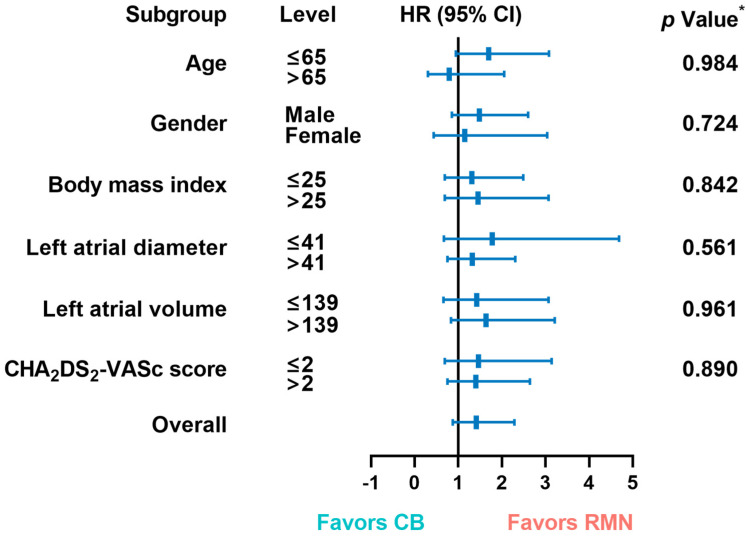
Hazard ratios for recurrence in patients in RMN and CB groups, in accordance with six clinical and demographic factors. Each rectangle indicates the estimated treatment effect and the horizontal lines represent the 95% confidence intervals. * *p*-Value from the interaction term in Cox regression model. HR: Hazard ratio; CI: Confidence interval; CB: Cryoballoon; RMN: Robotic magnetic navigation.

The mid-term success rates were 71% and 62% for patients assigned to the RMN and CB groups, respectively. As depicted in Figure 1, we compared the AF recurrence free-survival after 3 years of an initial ablation procedure between the two groups. The difference in ablation techniques did not produce significant variations in AF recurrence-free survival between the two groups (*p* = 0.158), which was consistent with the interval-censored Cox model (*p* = 0.166), as shown in Supplementary Figure S1. Next, we performed a pairwise subgroup analysis based on six clinical and demographic factors (Figure 2) and found no significant interaction between the ablation techniques and six factors.

### 3.3. Secondary Endpoint

Large differences were available in the periprocedural parameters between the two groups (Table 2). For intra-procedure radiation metrics, including fluoroscopy time and dose, the parameters of the CB group were almost twice those of the RMN group. However, regarding the parameters of procedure duration and left atrial procedure duration, the RMN group was significantly higher than the CB group (all *p* < 0.0001).

### 3.4. Cluster Analysis

**Figure 3:**
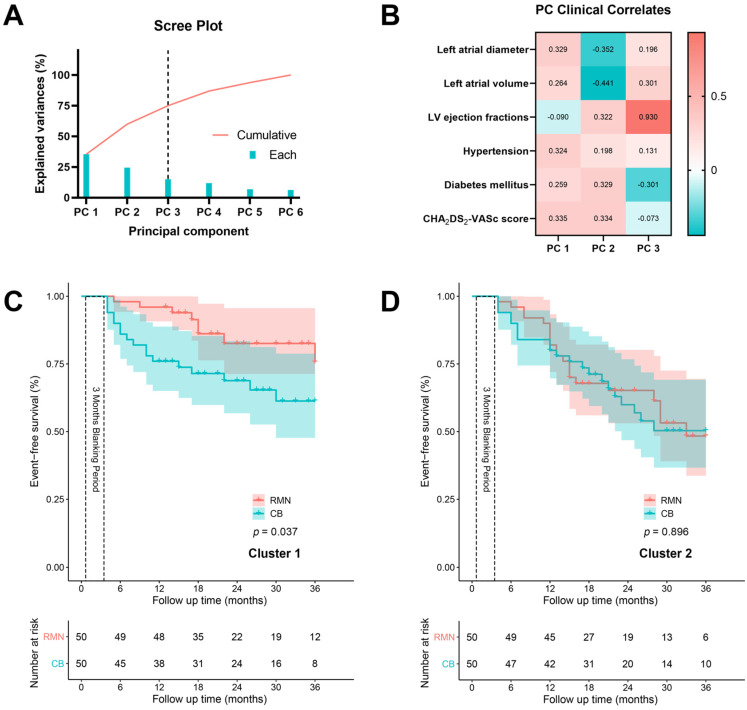
Kaplan–Meier curves of two clusters identified by the principal component and cluster analyses. (**A**) The scree plot showing the explained variances of each component, which is extracted from the principal component analysis. The first three principal components (74.9% cumulative variances explained) were adopted for further cluster analysis. (**B**) The heat map showing the relationship between the three identified principal components and clinical parameters. (**C**) Two clusters of patients were identified by the Two-step cluster analysis using the three principal components. The freedom from recurrence of AF differed significantly between the RMN and CB groups in cluster 1 (*p* = 0.037). (**D**) The freedom from recurrence of AF did not differ between the RMN and CB groups in cluster 2 (*p* = 0.896). PC: Principal component; LV: Left ventricular; RMN: Robotic magnetic navigation; CB: Cryoballoon.

To eliminate the interaction of the clinical indicators, we used the principal component analysis and selected three components (74.9% cumulative variances explained) for further cluster analysis (Figure 3A,B). The Two-step cluster analysis returned two clusters of 100 patients, whose characteristics were listed in Table 3. In cluster 1, the patients exhibited a higher percentage of diabetes mellitus, and relatively smaller left atria. The Kaplan–Meier curves indicating the AF recurrence free-survival of the two clusters are shown in Figure 3C,D. In cluster 2, the technology used for ablation had little effect on AF recurrence-free survival (*p* = 0.896). However, in cluster 1, the AF recurrence-free survival of patients in the RMN group was significantly higher than in the CB group (*p* = 0.037). Moreover, these results were consistent with the interval-censored Cox model, as shown in Supplementary Figure S2.

### 3.5. Predictors of AF Recurrence

**Figure 4:**
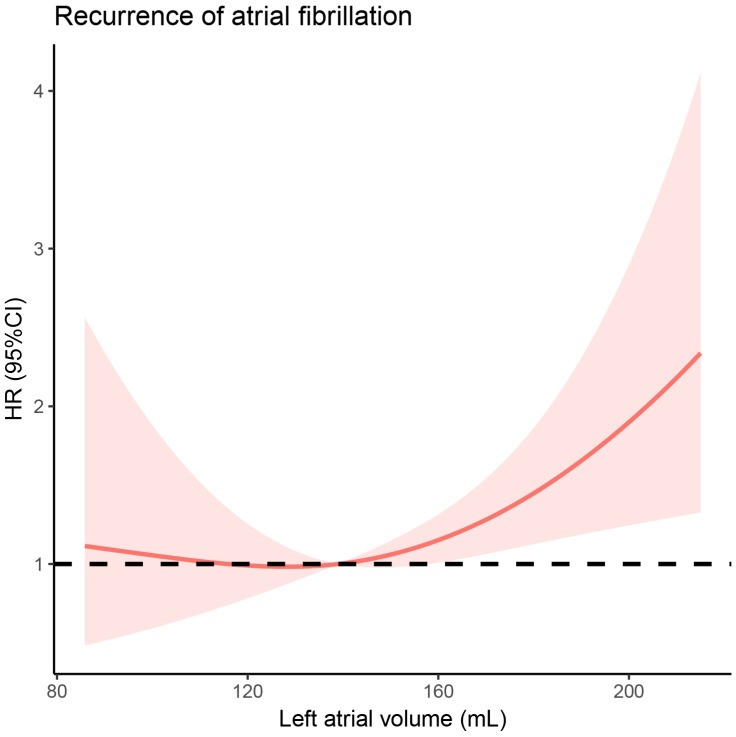
Adjusted hazard ratios of recurrence of atrial fibrillation, in accordance with the left atrial volume. Data were fitted using the Cox regression model of restricted cubic spline with three knots (10th, 50th, and 90th percentiles) and adjusted for patient age, gender, body mass index, left ventricular ejection fractions, history of diabetes mellitus, hypertension, and ablation technology. The solid line indicates the estimated hazard ratio and the shaded area represents the 95% confidence interval. Reference is the median left atrial volume (139) of patients in this study. The *p*-value for overall and non-linearity associations were 0.012 and 0.171. HR: Hazard ratio; CI: Confidence interval.

The Cox regression analysis was used to identify factors which are correlated to AF recurrence (Table 4). In the univariate analysis, no factor was found to be significantly predictive of AF recurrence. In the multivariable analysis, the following factors were included: hypertension, LVEF, LAV, and ablation technology. LAV significantly increased the risk of AF recurrence (adjusted hazard ratios = 1.007, *p* = 0.048). We further performed a Cox regression model of restricted cubic spline to visualize, on a continuous scale, the association between AF recurrence and patients’ LAV (Figure 4). The model was adjusted for patient age, gender, BMI, LVEF, history of diabetes mellitus, hypertension, and ablation technology. The spline was almost flat until approximately 139 of LAV, where the risk of AF recurrence began to rise rapidly (*p*-value for overall association = 0.012).

## 4. Discussion

### 4.1. Main Findings

For PerAF, this prospective, non-randomized, concurrent controlled trial provides initial insight into the comparison of mid-term outcomes and peri-procedure parameters between CB and RMN-guided ablation. Our main findings are as follows: CB is generally equivalent to RMN-guided ablation for patients with PerAF. However, RMN-guided ablation is favorable for specific patients, as evidenced by the mid-term outcomes in this study. Compared with CB, RMN-guided ablation leads to a longer procedure time, but with less radiation. LAV is an important prognostic factor and is significantly correlated with the risk of AF recurrence.

### 4.2. Mid-Term Outcomes

The mid-term success rate of CB and RMN-guided ablation for patients with PerAF was consistent with previous reports [11,12,13]. For PerAF, this is the first study that demonstrates the generally equivalent mid-term outcomes of these two distinctly different technologies. Of note, we identified a cluster of patients with a higher percentage of diabetes mellitus and relatively low LAV. RMN-guided ablation is favorable for this cluster of patients, as evidenced by the Kaplan−Meier curves of AF recurrence-free survival. Currently, the Kaplan−Meier method remains the primary way to access the recurrence of AF. However, mathematically or statistically, it may not be the ideal approach, since the time of recurrence recognition rarely represents the time of its initiation [14]. Therefore, we utilized the interval-censored Cox model to attest our results. Interestingly, the two approaches reached an agreement in the current study. Unlike RMN, the isolated area of CB was determined by the size of balloon, which was selected and used. The reduced long-term efficacy of CB might result from the confusion of ‘how to choose the optimal size of CB’. Previous studies have demonstrated that diabetes mellitus could contribute to changes of electrophysiological substrates that trigger and maintain PerAF [15]. RMN, a robot-assisted technology, provides operators with greater flexibility and choice in determining the location and size of ablation areas, which may be the reason for its better performance in this complex pathological setting. Extensive PV isolation, including PV carina, antrum, and LA posterior wall, have been reported to be associated with improved clinical outcomes [16]. This may result from the elimination of AF triggers or substrates in these non-PV areas. Kenigsberg et al. [17] found that the area of posterior LA wall ablation with the CB catheter is wide and antral. Only 27% of the posterior LA wall was intact after lesion placement. Therefore, posterior LA wall debulking by CB may explain the superiority of the outcome in patients with larger LA. In summary, we cannot conclude that RMN is superior to CB for PerAF in this study. However, RMN may be a better choice for patients presenting with clinical manifestations, as described above. Future multi-center, randomized trials may provide additional valuable data to the global EP community.

### 4.3. Peri-Procedure Parameters

The distinct differences in peri-procedure parameters between the CB and RMN groups may arise from the characteristics of RMN, which are listed in Table 5. The reduced procedure duration of CB will likely be ascribed to the ability of achieving PVI in a single or few treatment applications. Given its comparable long-term efficacy to RMN, CB may have a shorter learning curve and improve the utilization of catheterization laboratory, especially for physician operators without significant RF catheter skill or experience. On the other hand, increased fluoroscopy time and dose with CB remains a considerable and seemingly often overlooked metric that RMN addresses. Procedure duration is a potential limitation for RMN-guided ablation. However, this should be viewed differently than data pertaining to the manual or traditional pull-wire catheter RF treatment. Prolonged procedure duration with RMN usually does not cause fatigue [18] due to the control room remote-operation mode. Therefore, this does not lead to compromised efficacy or safety. Few studies have reported other limitations of RMN. The prevalence of claustrophobia is very low, and thus has little impact on the size of target population for RMN applications. Compared with manual ablation, there is no tactile feedback for RMN when the catheter tip contacts the atrial wall. However, contact force catheters may be capable of tackling this problem.

### 4.4. Predictors of AF Recurrence

Studies focusing on predictors of AF recurrence in patients with PerAF are few and exhibit inconsistent conclusions. In a previous systematic review that included both paroxysmal AF and PerAF, it was determined that LAD did not predict recurrence [19]. Conversely, previous guidelines cite several studies that regarded LAD as a potential predictor [20]. This seeming paradox may be the result of different statistical treatments that are applied to the included variables. In the current study, the use of Cox regression model with restricted cubic spline provided us with a way to perform spline modeling without human intervention on continuous variables, thus reducing bias. We identified LAV as a predictor for AF recurrence and demonstrated the association between AF recurrence and LAV on a continuous scale. We hypothesize that LAD may not be sufficient to represent LAV in all cases, leading to the apparent paradox. Moreover, the updated guide found that the LAV index (LAV corrected body surface area) was a great factor for AF recurrence prediction [21]. This is reasonable, and future more sophisticated radiographic tools, that assess the anatomy of the left atrium, may lead to a more accurate index. Interestingly, a recent meta-analysis [22], including both paroxysmal AF and PerAF patients, was consistent with the current study. This suggests similar recurrence mechanisms between paroxysmal AF and PerAF. Several preclinical studies [23,24] have demonstrated the pathophysiological role of LA remodeling in promoting AF, although it has not been determined whether the enlarged LA size is a cause or consequence of AF.

### 4.5. Limitations

Of note, this is not a typical randomized, controlled trial. However, patients were self-selected into groups on the basis of their willingness. Due to unwillingness of the patients or clinical follow-up protocol, implantable continuous monitoring of heart rhythm was not feasible in the present study. The employed Kaplan-Meier analysis based on intermittent Holter-ECG may not necessarily reflect the exact characteristics of arrhythmia recurrence in both groups. Nonetheless, Holter-ECG currently remains an important method to assess the arrhythmia recurrence for patients in whom continuous heart rhythm monitoring is not available. The initial comparison on mid-term outcomes and peri-procedure parameters of CB and RMN-guided ablation for PerAF needs to be confirmed by further multi-center, randomized, controlled trials. Investigation of safety profiles between the two techniques is not included and beyond the scope of this study, but may be of interest for further studies.

## 5. Conclusions

In general, CB is equivalent to RMN-guided ablation for patients with PerAF. However, RMN-guided ablation is favorable for patients with diabetes mellitus and a relatively low LAV.

## Figures and Tables

**Table 1:** Baseline characteristics.

	Total	RMN	CB	*p*-Value
	*n* = 200	*n* = 100	*n* = 100	
Age, years	59.1 ± 9.8	60.3 ± 10.1	57.9 ± 9.4	0.091
Male, *n* (%)	150 (75)	77 (77)	73 (73)	0.514
BMI, kg/m^2^	25.2 ± 3.1	25.5 ± 3.0	24.9 ± 3.2	0.147
Hypertension, *n* (%)	101 (51)	54 (54)	47 (47)	0.322
Diabetes mellitus, *n* (%)	25 (13)	14 (14)	11 (11)	0.521
Stroke/TIA/thrombo-embolism, *n* (%)	9 (5)	3 (3)	6 (6)	0.498
Vascular disease, *n* (%)	103 (52)	53 (53)	50 (50)	0.671
LVEF, %	63.7 ± 6.1	63.7 ± 5.9	63.8 ± 6.3	0.934
LAD, mm	43.1 ± 3.6	43.1 ± 3.1	43.1 ± 4.0	0.966
LAV, mL	143.4 ± 38.4	142.6 ± 37.3	144.1 ± 39.6	0.796
CHA_2_DS_2_-VASc score	1.9 ± 1.4	1.9 ± 1.4	1.8 ± 1.3	0.345
0, *n* (%)	31 (16)	14 (14)	17 (17)	-
1, *n* (%)	59 (30)	28 (28)	31 (31)	-
2, *n* (%)	54 (27)	27 (27)	27 (27)	-
3–5, *n* (%)	56 (28)	31 (31)	25 (25)	-

RMN: Robotic magnetic navigation; CB: Cryoballoon; BMI: Body mass index; TIA: Transient ischemic attack; LVEF: Left ventricular ejection fractions; LAD: Left atrial diameter; LAV: Left atrial volume.

**Table 2:** Procedural parameters.

	Total	RMN	CB	*p*-Value
	*n* = 200	*n* = 100	*n* = 100	
Fluoroscopy time, mins	10.5 ± 5.9	6.8 ± 3.1	13.9 ± 5.9	<0.0001
Fluoroscopy dose, mGy	243.4 ± 190.7	135.6 ± 84.2	342.4 ± 207.2	<0.0001
Fluoroscopy dose, μGym^2^	2697.2 ± 2186.1	1459.1 ± 1193.3	3849.0 ± 2273.4	<0.0001
Procedure duration, mins	108.1 ± 37.4	136.0 ± 29.1	83.5 ± 24.6	<0.0001
LA procedure duration, mins	90.6 ± 37.9	118.8 ± 29.3	65.7 ± 25.0	<0.0001

RMN: Robotic magnetic navigation; CB: Cryoballoon; LA: Left atrial.

**Table 3:** Clinical manifestations of patients in the two clusters.

	Cluster 1			Cluster 2			*p*-Value
	Total *n* = 100	RMN *n* = 50	CB *n* = 50	Total *n* = 100	RMN *n* = 50	CB *n* = 50	
Age, years	58.5 ± 11.1	59.5 ± 11.6	57.5 ± 10.7	59.7 ± 8.2	61.0 ± 8.3	58.3 ± 8.0	0.395
Male, *n* (%)	73 (73)	42 (84)	31 (62)	77 (77)	35 (70)	42 (84)	0.514
BMI, kg/m^2^	24.8 ± 3.2	25.1 ± 3.1	24.5 ± 3.4	25.6 ± 2.9	25.9 ± 2.9	25.3 ± 2.9	0.090
Hypertension, *n* (%)	44 (44)	24 (48)	20 (40)	57 (57)	30 (60)	27 (54)	0.066
Diabetes mellitus, *n* (%)	23 (23)	14 (28)	9 (18)	2 (2)	0 (0)	2 (4)	<0.0001
Stroke/TIA/thrombo-embolism, *n* (%)	2 (2)	0 (0)	2 (4)	7 (7)	3 (6)	4 (8)	0.170
Vascular disease, *n* (%)	46 (46)	25 (50)	21 (42)	57 (57)	28 (56)	29 (58)	0.157
LVEF ≤ 50%, *n* (%)	5 (5)	2 (4)	3 (6)	0 (0)	0 (0)	0 (0)	0.087
LAD, mm	42.0 ± 3.8	42.1 ± 3.4	41.8 ± 4.1	44.2 ± 3.0	44.1 ± 2.5	44.3 ± 3.4	<0.0001
LAV, ml	128.0 ± 33.3	127.2 ± 34.2	128.7 ± 32.8	159.1 ± 37.1	159.1 ± 33.6	159.1 ± 40.3	<0.0001
CHA_2_DS_2_-VASc score	1.8 ± 1.5	1.9 ± 1.6	1.7 ± 1.5	1.9 ± 1.2	2.0 ± 1.1	1.8 ± 1.2	0.230

BMI: Body mass index; TIA: Transient ischemic attack; LVEF: Left ventricular ejection fractions; LAD: Left atrial diameter; LAV: Left atrial volume. No significant difference was observed within the cluster.

**Table 4:** Cox regression analysis of prognostic factors.

	Univariate Analysis		Multivariate Analysis	
	HR (95% CI)	*p*-Value	HR (95% CI)	*p*-Value
Age	1.000 (0.976–1.025)	0.980		
Male	0.964 (0.556–1.671)	0.896		
BMI	0.979 (0.903–1.061)	0.598		
Hypertension	1.480 (0.910–2.406)	0.114	1.244 (0.744–2.081)	0.405
Diabetes mellitus	0.736 (0.318–1.705)	0.475		
Stroke/TIA/thrombo-embolism	0.561 (0.137–2.291)	0.421		
Vascular disease	0.911 (0.564–1.471)	0.703		
LVEF	1.029 (0.987–1.072)	0.180	1.022 (0.980–1.066)	0.312
LAD	0.982 (0.917–1.053)	0.613		
LAV	1.006 (1.000–1.013)	0.057	1.007 (1.000–1.013)	0.048
CHA_2_DS_2_-VASc score	1.004 (0.837–1.205)	0.964		
CB	1.411 (0.870–2.288)	0.163	1.565 (0.930–2.633)	0.092
CHA2DS2-VASc score × ln(time)	1.021 (0.951–1.097)	0.564		

BMI: Body mass index; TIA: Transient ischemic attack; RMN: Robotic magnetic navigation; LVEF: Left ventricular ejection fractions; LAD: Left atrial diameter; LAV: Left atrial volume, CB: Cryoballoon.

**Table 5:** Overview of the RMN-guided ablation.

	Characteristics
Major benefits	flexible and precise
	stable focal contact
	reduced radiation
	reduced physician fatigue
	better patient safety
Potential limitations	prolonged procedure time
	special contraindications: claustrophobia
	no tactile feedback

## Data Availability

The data presented in this study are available on reasonable request.

## Supplementary Materials

The following supporting information can be downloaded at: https://www.mdpi.com/article/10.3390/jcdd9030088/s1, Table S1: Proportional hazards assumption for Kaplan-Meier method; Table S2: Proportional hazards assumption for univariate Cox regression analysis; Table S3: Proportional hazards assumption for multivariate Cox regression analysis; Figure S1: Interval-censored Cox model showing the cumulative freedom from recurrence of atrial fibrillation after RMN-guided ablation and CB ablation; Figure S2: Interval-censored Cox model of the two clusters showing the cumulative freedom from recurrence of atrial fibrillation after RMN-guided ablation and CB ablation.

## References

[1] Vinter N., Huang Q., Fenger-Grøn M., Frost L., Benjamin E.J., Trinquart L. (2020). Trends in excess mortality associated with atrial fibrillation over 45 years (Framingham Heart Study): Community based cohort study. BMJ.

[2] Schnabel R.B., Yin X., Gona P., Larson M.G., Beiser A., McManus D.D., Newton-Cheh C., Lubitz S.A., Magnani J.W., Ellinor P. (2015). 50 year trends in atrial fibrillation prevalence, incidence, risk factors, and mortality in the Framingham Heart Study: A cohort study. Lancet.

[3] Miyasaka Y., Barnes M.E., Gersh B.J., Cha S.S., Bailey K.R., Abhayaratna W.P., Seward J.B., Tsang T.S. (2006). Secular Trends in Incidence of Atrial Fibrillation in Olmsted County, Minnesota, 1980 to 2000, and Implications on the Projections for Future Prevalence. Circulation.

[4] Krijthe B.P., Kunst A., Benjamin E., Lip G.Y., Franco O., Hofman A., Witteman J.C., Stricker B.H., Heeringa J. (2013). Projections on the number of individuals with atrial fibrillation in the European Union, from 2000 to 2060. Eur. Heart J..

[5] Wilber D.J., Pappone C., Neuzil P., De Paola A., Marchlinski F., Natale A., Macle L., Daoud E.G., Calkins H., Hall B. (2010). Comparison of antiarrhythmic drug therapy and radiofrequency catheter ablation in patients with paroxysmal atrial fibrillation: A randomized controlled trial. Jama.

[6] Kuck K.-H., Brugada J., Fürnkranz A., Metzner A., Ouyang F., Chun K.J., Elvan A., Arentz T., Bestehorn K., Pocock S.J. (2016). Cryoballoon or Radiofrequency Ablation for Paroxysmal Atrial Fibrillation. N. Engl. J. Med..

[7] Bhaskaran A., Barry M.A., Al Raisi S.I., Chik W., Nguyen D.T., Pouliopoulos J., Nalliah C., Hendricks R., Thomas S., McEwan A.L. (2015). Magnetic guidance versus manual control: Comparison of radiofrequency lesion dimensions and evaluation of the effect of heart wall motion in a myocardial phantom. J. Interv. Card. Electrophysiol..

[8] Qian P., De Silva K., Kumar S., Nadri F., Samanta R., Bhaskaran A., Ross D., Sivagangabalan G., Cooper M., Kizana E. (2018). Early and long-term outcomes after manual and remote magnetic navigation-guided catheter ablation for ventricular tachycardia. EP Eur..

[9] Kuck K.-H., Fürnkranz A., Chun K.J., Metzner A., Ouyang F., Schlüter M., Elvan A., Lim H.W., Kueffer F.J., Arentz T. (2016). Cryoballoon or radiofrequency ablation for symptomatic paroxysmal atrial fibrillation: Reintervention, rehospitalization, and quality-of-life outcomes in the Fire and Ice trial. Eur. Heart J..

[10] Li X., Jin Q., Zhang N., Ling T., Lin C., Jia K., Bao Y., Xie Y., Wei Y., Chen K. (2020). Procedural outcomes and learning curve of cardiac arrhythmias catheter ablation using remote magnetic navigation: Experience from a large-scale single-center study. Clin. Cardiol..

[11] Tondo C., Iacopino S., Pieragnoli P., Molon G., Verlato R., Curnis A., Landolina M., Allocca G., Arena G., Fassini G. (2018). Pulmonary vein isolation cryoablation for patients with persistent and long-standing persistent atrial fibrillation: Clinical outcomes from the real-world multicenter observational project. Heart Rhythm.

[12] Lemes C., Wissner E., Lin T., Mathew S., Deiss S., Rillig A., Heeger C., Wohlmuth P., Reissmann B., Tilz R. (2016). One-year clinical outcome after pulmonary vein isolation in persistent atrial fibrillation using the second-generation 28 mm cryoballoon: A retrospective analysis. EP Eur..

[13] Muntean B., Gutleben K.-J., Heintze J., Vogt J., Horstkotte D., Nölker G. (2012). Magnetically guided irrigated gold-tip catheter ablation of persistent atrial fibrillation—techniques, procedural parameters and outcome. J. Interv. Card. Electrophysiol..

[14] Shemin R.J., Cox J.L., Gillinov A.M., Blackstone E.H., Bridges C.R. (2007). Guidelines for Reporting Data and Outcomes for the Surgical Treatment of Atrial Fibrillation. Ann. Thorac. Surg..

[15] Wang A., Green J.B., Halperin J.L., Piccini J.P. (2019). Atrial Fibrillation and Diabetes Mellitus. J. Am. Coll. Cardiol..

[16] Lin Y.-J., Tsao H.-M., Chang S.-L., Lo L.-W., Tuan T.-C., Hu Y.-F., Tsai W.-C., Chang C.-J., Tai C.-T., Suenari K. (2011). The distance between the vein and lesions predicts the requirement of carina ablation in circumferential pulmonary vein isolation. EP Eur..

[17] Kenigsberg D.N., Martin N., Lim H.W., Kowalski M., Ellenbogen K.A. (2015). Quantification of the cryoablation zone demarcated by pre- and postprocedural electroanatomic mapping in patients with atrial fibrillation using the 28-mm second-generation cryoballoon. Heart Rhythm.

[18] Bassil G., Markowitz S.M., Liu C.F., Thomas G., Ip J.E., Lerman B.B., Cheung J.W. (2020). Robotics for catheter ablation of cardiac arrhythmias: Current technologies and practical approaches. J. Cardiovasc. Electrophysiol..

[19] Balk E.M., Garlitski A.C., Alsheikh-Ali A.A., Terasawa T., Chung M., Ip S. (2010). Predictors of Atrial Fibrillation Recurrence After Radiofrequency Catheter Ablation: A Systematic Review. J. Cardiovasc. Electrophysiol..

[20] Kirchhof P., Benussi S., Kotecha D., Ahlsson A., Atar D., Casadei B., Castella M., Diener H.C., Heidbuchel H., Hendriks J. (2016). 2016 ESC Guidelines for the management of atrial fibrillation developed in collaboration with EACTS. EP Eur..

[21] Hindricks G., Potpara T., Dagres N., Arbelo E., Bax J.J., Blomström-Lundqvist C., Boriani G., Castella M., Dan G.-A., Dilaveris P.E. (2021). Corrigendum to: 2020 ESC Guidelines for the diagnosis and management of atrial fibrillation developed in collaboration with the European Association for Cardio-Thoracic Surgery (EACTS): The Task Force for the diagnosis and management of atrial fibrillation of the European Society of Cardiology (ESC) Developed with the special contribution of the European Heart Rhythm Association (EHRA) of the ESC. Eur. Heart J..

[22] Njoku A., Kannabhiran M., Arora R., Reddy P., Gopinathannair R., Lakkireddy D., Dominic P. (2018). Left atrial volume predicts atrial fibrillation recurrence after radiofrequency ablation: A meta-analysis. EP Eur..

[23] Ge Z., Chen Y., Wang B., Zhang X., Yan Y., Zhou L., Zhang Y., Xie Y. (2020). MFGE8 attenuates Ang-II-induced atrial fibrosis and vulnerability to atrial fibrillation through inhibition of TGF-β1/Smad2/3 pathway. J. Mol. Cell Cardiol..

[24] Thomas L., Abhayaratna W.P. (2017). Left Atrial Reverse Remodeling: Mechanisms, Evaluation, and Clinical Significance. JACC Cardiovasc. Imaging.

