# Supplementary material for: Comparison of the Mid-Term Outcomes of Robotic Magnetic Navigation-Guided Radiofrequency Ablation versus Cryoballoon Ablation for Persistent Atrial Fibrillation
Source: J Cardiovasc Dev Dis. 2022 Mar 17;9(3):88. doi: 10.3390/jcdd9030088 (PMC8953767; doi:10.3390/jcdd9030088)
Supplement: Supplementary file 1 [file jcdd-09-00088-s001.zip › jcdd-1561894-supplementary.pdf]

Table S1. Proportional hazards assumption for Kaplan-Meier method

	***p* value**
Group (in all patients)	0.0996
Group (in Cluster1)	0.1096
Group (in Cluster2)	0.5686

Table S2. Proportional hazards assumption for univariate Cox regression analysis

	***p* value**
Age	0.0602
Male	0.9373
BMI	0.4909
Hypertension	0.9155
Diabetes mellitus	0.4727
Stroke/TIA/thrombo-embolism	0.5282
Vascular disease	0.0750
LVEF	0.0639
LAD	0.5502
LAV	0.2854
CHA_2DS_2-VASc score	0.0473*
CB	0.0996

BMI, body mass index; TIA, transient ischemic attack; LVEF, left ventricular ejection fractions; LAD, left atrial diameter; LAV, left atrial volume, CB, cryoballoon.

Table S3. Proportional hazards assumption for multivariate Cox regression analysis

	***p* value**
Hypertension	0.8951
LVEF	0.1046
LAV	0.2437
CB	0.0949
Global test	0.1943

LVEF, left ventricular ejection fractions; LAV, left atrial volume, CB, cryoballoon.

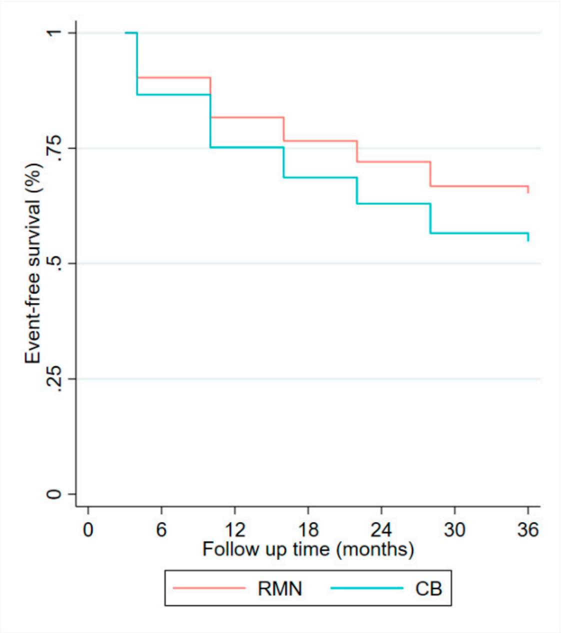

Figure S1. Interval-censored Cox model showing the cumulative freedom from recurrence of atrial fibrillation after RMN-guided ablation and CB ablation. The freedom from recurrence of AF did not differ between the 2 groups (p = 0.166). RMN = robotic magnetic navigation; CB = cryoballoon.

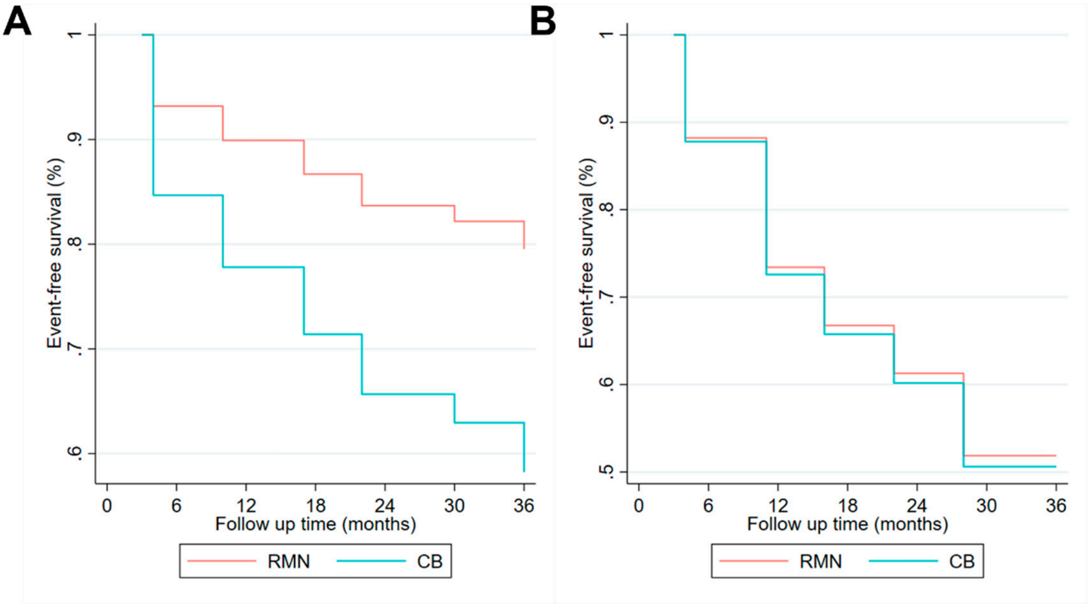

Figure S2. Interval-censored Cox model of the two clusters showing the cumulative freedom from recurrence of atrial fibrillation after RMN-guided ablation and CB ablation. The freedom from recurrence of AF differed significantly between RMN and CB group in cluster 1 ($p < 0.05$) (A), but not in cluster 2 (p = 0.905) (B). RMN = robotic magnetic navigation; CB = cryoballoon.
